# Lyophilized plasma attenuates vascular permeability, inflammation and lung injury in hemorrhagic shock

**DOI:** 10.1371/journal.pone.0192363

**Published:** 2018-02-02

**Authors:** Shibani Pati, Zhanglong Peng, Katherine Wataha, Byron Miyazawa, Daniel R. Potter, Rosemary A. Kozar

**Affiliations:** 1 Department of Laboratory Medicine, University of California San Francisco, San Francisco, California, United States of America; 2 Department of Anesthesia, University of Texas Health Science Center at Houston, Houston, Texas, United States of America; 3 Shock Trauma Center, University of Maryland School of Medicine, Baltimore, Maryland, United States of America; Medical College of Georgia, Augusta, UNITED STATES

## Abstract

In severe trauma and hemorrhage the early and empiric use of fresh frozen plasma (FFP) is associated with decreased morbidity and mortality. However, utilization of FFP comes with the significant burden of shipping and storage of frozen blood products. Dried or lyophilized plasma (LP) can be stored at room temperature, transported easily, reconstituted rapidly with ready availability in remote and austere environments. We have previously demonstrated that FFP mitigates the endothelial injury that ensues after hemorrhagic shock (HS). In the current study, we sought to determine whether LP has similar properties to FFP in its ability to modulate endothelial dysfunction in vitro and in vivo. Single donor LP was compared to single donor FFP using the following measures of endothelial cell (EC) function *in vitro*: permeability and transendothelial monolayer resistance; adherens junction preservation; and leukocyte-EC adhesion. *In vivo*, using a model of murine HS, LP and FFP were compared in measures of HS- induced pulmonary vascular inflammation and edema. Both *in vitro* and *in vivo* in all measures of EC function, LP demonstrated similar effects to FFP. Both FFP and LP similarly reduced EC permeability, increased transendothelial resistance, decreased leukocyte-EC binding and persevered adherens junctions. *In vivo*, LP and FFP both comparably reduced pulmonary injury, inflammation and vascular leak. Both FFP and LP have similar potent protective effects on the vascular endothelium in vitro and in lung function in vivo following hemorrhagic shock. These data support the further development of LP as an effective plasma product for human use after trauma and hemorrhagic shock.

## Introduction

Globally, it is estimated there are over 5 million deaths per year due to traumatic injury, many of which occur from uncontrolled bleeding. [1] Hemorrhage in fact remains the number one cause of early trauma deaths and the primary cause of potentially preventable deaths in both military and civilian settings. In recent years, employing modern resuscitation strategies, which include the early, and empiric use of plasma in a balanced ratio with packed red cells have reduced early mortality. [2–8] This decrease in mortality is thought to be more than just a reduction in bleeding related deaths and is hypothesized to involve the protective effects of plasma to an injured and impaired endothelium, termed the “endotheliopathy of trauma (EoT).” [9,10] Clinically this is manifested as a pro-inflammatory state with vessel hyperpermability and tissue edema, all of which can result in organ failures and late deaths.

While the use of plasma has demonstrated benefits on outcome, little is known about its mechanism of action. Over the past few years pre-clinical and clinical studies have suggested that the mechanism may not be solely related to hemostasis, but also due in part to its ability to globally promote systemic vascular stability, defined by decreased endothelial cell (EC) permeability, decreased inflammation in the lungs and systemically as well as improved hemodynamic stabilization following hemorrhagic shock. [10–18] Plasma resuscitation in a hemorrhagic shock rodent model has also been shown to partially restore a damaged endothelial glycocalyx, including syndecan-1, the structural backbone of the glycocalyx [14,17,19]. Restoration of the glycocalyx may improve outcomes after trauma and hemorrhagic shock by attenuating leukocyte-EC adhesion, platelet adhesion, and inflammatory cytokine binding by establishing a physical barrier between the blood and EC barrier. [19–25] Plasma and plasma products have been shown to decrease endothelial cell permeability, inflammation, and lung injury both *in vitro* and *in vivo* and may contribute to improved outcomes [10–18].

Despite its benefits, the use of fresh frozen plasma (FFP) in traumatic injury comes with a significant logistical challenge and the burden of shipping and storage of frozen blood products around the world. Conversely, lyophilized plasma (LP) can be stored at room temperature, transported easily, and reconstituted rapidly in remote and austere environments. [26] Due to its rapid reconstitution time, LP can be administered much earlier than FFP and is ideal for pre-hospital use where transport times are lengthened due to location and logistical challenges. Furthermore, the storage lesion that develops in thawed plasma would not occur with LP, as only what is needed is reconstituted. Once thawed, fresh frozen plasma is approved for use for up to five days when stored at 4°C. Our group has shown that the beneficial effects of FFP on EC function and hemodynamic stability *in vitro* and *in vivo* decrease over time from day zero thru day five of storage. [27] Our collaborators have also shown that an increase in TGF-β in day 5 FFP inhibits endothelial cell migration, which potentially results in diminished repair of injured vessels and tissue during trauma and HS. [12] There is considerable interest therefore to develop dried plasma products in the US that have expanded storage capabilities and can be rapidly reconstituted. In addition, pathogen reduction is feasible for dried products and can potentially reduce the risk of infection of new emerging pathogens and transfusion transmitted infections [26]. We therefore sought to determine if lyophilized plasma would demonstrate comparable protection to FFP by examining endothelial cell function in vitro and lung function in vivo in a clinically relevant mouse model of hemorrhagic shock.

## Materials and methods

### Plasma

Fresh Frozen Plasma (FFP) type male O+ was obtained from Gulf Coast Regional Blood Center and from Blood Center’s of the Pacific (BCP). Lyophilized type male O+ plasma was obtained from the HEMOCON (Portland,OR) and reconstituted in buffer provided by Hemcon (Portland, OR). The lyophilized plasma utilized in the current study tested was produced as part of a contract from the US Army. The program made significant progress in optimizing conditions, resulting in a process that yielded factor levels within the normal range, The product proceeded to and was successful in a phase I clinical trial. [28] However, the company is no longer in business, but the same technology is currently being developed by Vascular Solutions (Maple Grove, Minnesota) which is part of Teleflex (Wayne, PA). It is expected that this program will produce a FDA approved product for US use by 2020–2021. [26]

### *In vitro* studies

#### Primary cells and cell lines

Early passage Human Umbilical Vein Endothelial Cells (HUVECS) were purchased from Lonza (Walkersville, MD). Cells were maintained in EGM-2MV media (Lonza) and used for in-vitro VE-Cadherin and B-catenin staining and flow cytometry. U937 cells were obtained from ATCC (Bethesda, MD) and passaged in RPMI with 10% fetal bovine serum. All experiments were conducted in U937 between passages 3–8. All cell lines were maintained at 37°C and 5% CO_2_ in a humidified incubator.

#### Resuscitation fluids

Treatment groups tested *in vitro* were 1) FFP (Day 0 freshly thawed) 2) LP 3) Lactated Ringers Solution (LR) and 4) no treatment (media alone). All fluids were diluted to 10% or 30% into EC media- EGM-2MV. We have previously shown 10% dilution to be effective. [12,16]

#### Endothelial cell (EC) permeability

Collagen-coated 0.4μm pore size inserts were obtained from BD Biosciences. ECs were seeded at 40,000 cells per insert well in a total volume of 500μl of EGM-2 MV media and cultured for 8 hours to allow for cell attachment and adhesion. When confluence was reached, monolayers of ECs in the transwells were pre-treated with the five treatment groups (FFP, LP, LR and media control) for one hour at 10 and 30% total well volume. Permeability was then induced in monolayers with 50ng/ml VEGF-A165 from R&D (Minneapolis, MN), administered simultaneously with the addition of FITC-Dextran. EC monolayer permeability was tested by adding 50μl of 2mg/mL 40 kDa dextran conjugated to alexa fluor 480 (Sigma-Aldrich, St. Louis, MO) to the upper chamber of each well (final concentration). 75 μl samples were removed at timed intervals from the bottom well to determine the amount of fluorescent signal that had passed through the monolayer, which directly correlates to the degree of paracellular permeability in each sample. Measurements were determined with a fluorimeter (Biotek Synergy Biotek Winooski, VT) using excitation and emission wavelengths of 485nm and 530nm, respectively, from samples obtained 45 minutes after addition of FITC-Dextran. This is the same experiment that was run in Wataha et al. (2013) [16]

#### Endothelial Cell Impedance Assays (ECIS)

Endothelial cell barrier function was assessed as described in Potter et al. (2015) [18]. Briefly, trans-endothelial electrical resistance (TEER) is measured by ECIS system (ECIS 1600, Applied BioPhysics, Troy, NY) Confluent HUVECs were treated with 10% FFP, LP, LR or media alone and TEER measured at 4/16/64 kHz continuously for 2.5 hours afterward (n = 6 /group). Resistance measurements were expressed as the area under the curve for 2 hours.

#### In vitro VE-cadherin and β-catenin staining

ECs at passage 3 were seeded into 8-well glass chambered slides and grown for 48 hours at 37°C. The cells were treated with 10% dilutions of the groups diluted into EGM2-MV basal media for 1 hour, and then stimulated with VEGF-A (50 ng/ml) for 30 minutes (R&D Systems #293-VE-010, Minneapolis, MN). The cells were then fixed in 2% formaldehyde and blocked in TBS-T + 2.5% normal goat serum for 60 minutes at room temperature. A rabbit α-VE-cadherin antibody (1:400 dilution, Cell Signaling #2500 Beverly, MA), and a mouse α-β-catenin antibody (1:200 dilution, Cell Signaling #2677) were applied overnight at 4°C and detected using an Alexa 488 α-rabbit antibody (1:500 dilution, Molecular Probes #A-11034, Invitrogen, Carlsbad, CA) and an Alexa 568 α-mouse antibody (1:500 dilution, Invitrogen Molecular Probes #A-11031, Invitrogen). The next day the slides were mounted using ProLong Gold anti-fade reagent with DAPI (Molecular Probes P-36931, Invitrogen) to obtain nuclear staining. Images of the cells were taken at 40x on a Nikon A1R confocal microscope (Nikon Instruments, Inc, Melville, NY). Merged images (yellow) depict the degree of co-staining of VE-Cadherin and β-catenin indicating adherens junctions (AJs) mobilized at the endothelial cell membranes.

#### Leukocyte binding assays

ECs were grown to confluence on 96-well plates. 10^4^ cells/well were seeded and incubated at 37°C for two days or until confluent. Cells were pre-treated with the treatment groups for 1 hour at 10 and 30% plasma product of total well volume. As we have previously described, adhesion molecule expression was stimulated by the addition of TNFα (50ng/ml) for 4 hours following pre-treatment of the cells. [16] U937 cells were fluorescently labeled with Calcein-AM (Invitrogen Carlsbad, CA). A total of 10^4^ U937 cells, a well-established monocyte line, were added to wells and allowed to adhere for one hour. Non-adherent cells were gently washed away (3 washes) in PBS and labeled cells that remained bound to the ECs were quantified by fluorescent readings on the Biotek Analyzer. This is the same experiment that was run in Wataha et al. (2013) [16]

### In vivo studies

#### Rodent model of hemorrhagic shock

All procedures performed were protocols approved by the University of Texas Houston Medical School’s Institutional Animal Care and Use Committee (**IACUC)**. The experiments were conducted in compliance with the National Institutes of Health guidelines on the use of laboratory animals. All animals were housed at constant room temperature with a 12:12-h light-dark cycle with access to food and water ad libitum. We used our established coagulopathic mouse model of trauma-hemorrhagic shock. [15,18] Male C57BL/6J mice, 20–25 grams, underwent isoflurane anesthesia. A midline laparotomy incision was made, the organs and small bowel inspected, and then the incision closed. Bilateral femoral arteries were cannulated for continuous hemodynamic monitoring and blood withdrawal or resuscitation. Mice were bled to a mean arterial pressure (MAP) of 35±5 mmHg and maintained for 90 minutes. At the completion of the shock period, mice were resuscitated with either LR at 3x shed blood volume or FFP or LP at 1x shed blood volume. Mice will be awoken from anesthesia at the conclusion of resuscitation then underwent euthanasia by CO_2_ inhalation confirmed by cervical dislocation at 3 hours as we have found this time optimal to study organ function.[9]

#### Lung inflammation and histopathology

Lung inflammation was assessed by myeloperoxidase (MPO) immunostaining as in indicator of neutrophil influx. Paraffin-embedded tissue was cut into 5 μm-thick sections then incubated with MPO primary antibody (1:100 MPO mouse monoclonal antibody; Abcam, Cambridge, Mass) followed by incubation with secondary antibody (goat antiY-mouse; Alexa Fluor 568; Invitrogen). Two random images were taken from each lung section with a fluorescent microscope (Nikon Eclipse Ti, Melville, NY) at 200X magnification and immunofluorescence quantified using ImageJ software (National Institutes of Health). Results are expressed as relative fluorescent units (RFU). Sectioned tissue was also stained with hematoxylin and eosin (H&E) then scored by a blinded examiner for alveolar thickness, capillary congestion, and cellularity as originally described by Hart et al. and as we have employed. [14,29,30]

#### Lung vascular permeability

To measure Evans blue dye extravasation, animals received an intravenous injection of 3% Evans blue (4 mL/kg) two hours after the completion of resuscitation. One hour later, at the time of sacrifice, animals were perfused via right ventricle with 4°C PBS for 10 minutes to remove intravascular dye followed by 4% paraformadehyde at 4°C. Lungs were harvested then incubated in N-methylformamide for 24 h at 55°C to allow for dye extraction. After centrifugation, absorbance was measured in the supernatant at 620 nm using the VersaMax plate reader (Molecular Devices Inc, Sunnyvale, CA).

#### Lung edema

Lung tissue was weighed and then dried to constant weight at 50°C for 72 hours. The ratio of wet-to-dry was calculated by dividing the wet weight by the dry weight.

#### Statistical analysis

All data were analyzed by one-way analysis of variance (ANOVA) with Tukey’s test for post-hoc analysis with p<0.05. Data are expressed as mean ± SEM. *In vitro* data was repeated in triplicates unless indicated and for in vivo studies, n = at least 8/group.

## Results

### FFP and LP decrease endothelial permeability in vitro

**Fig 1:**
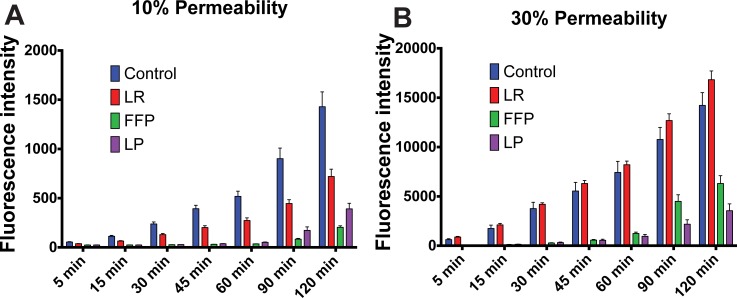
LP and FFP attenuate EC permeability on Transwells. Transwell permeability of treated wells to FITC-dextrans (40kD) after treatment with 10%(A) and 30%(B) of the fluids tested respectively. FFP and LP are significantly less than control and LR at time points 30 minutes and beyond while FFP and LP are significantly different on at the 90 and 120 time points, p < 0.05 via post hoc turkey tests of an unpaired Two-way ANOVA. Controls and FFP were featured in Wataha et al. (2013). [16].

**Fig 2:**
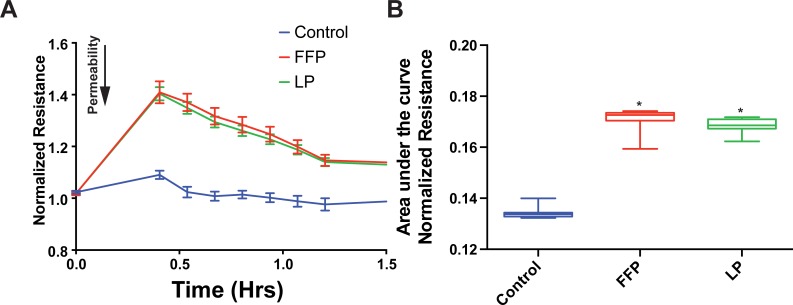
LP and FFP increase TEER of EC monolayers. (A) Mean average ECIS generated traces of the TEER of HUVECs treated with 10% media (control), LR, FFP or LP. (B) Area under the curve analysis for thirty minutes after the addition of treatment. * = (p<0.05) compared to control via post hoc turkey tests of an unpaired one-way ANOVA.

We have previously shown that FFP and pooled, solvent detergent treated spray dried plasma decreased endothelial cell permeability *in vitro*. [16] In the current study, we sought to determine if LP had similar effects on endothelial permeability. Fig 1A and 1B demonstrate that both LP and FFP attenuate EC permeability to FITC conjugated-dextran tracers over time compared to LR and media alone at concentrations of 10% and 30% of the total fluid volume. In support of these findings are the data shown in Fig 2 that EC monolayer resistance is similarly increased by FFP and LP depicted as the raw traces in (Fig 2A) and the bar graph depictions of total area under the curve (Fig 2A). Monolayer electrical resistance is directly correlated to the permeability of an endothelial monolayer. [31,32] Increased resistance indicates decreased permeability. These results both support the premise that FFP and LP equivalently attenuate EC permeability and monolayer barrier dysfunction.

### FFP and LP preserve EC adherens junctions on activated endothelial cells

**Fig 3:**
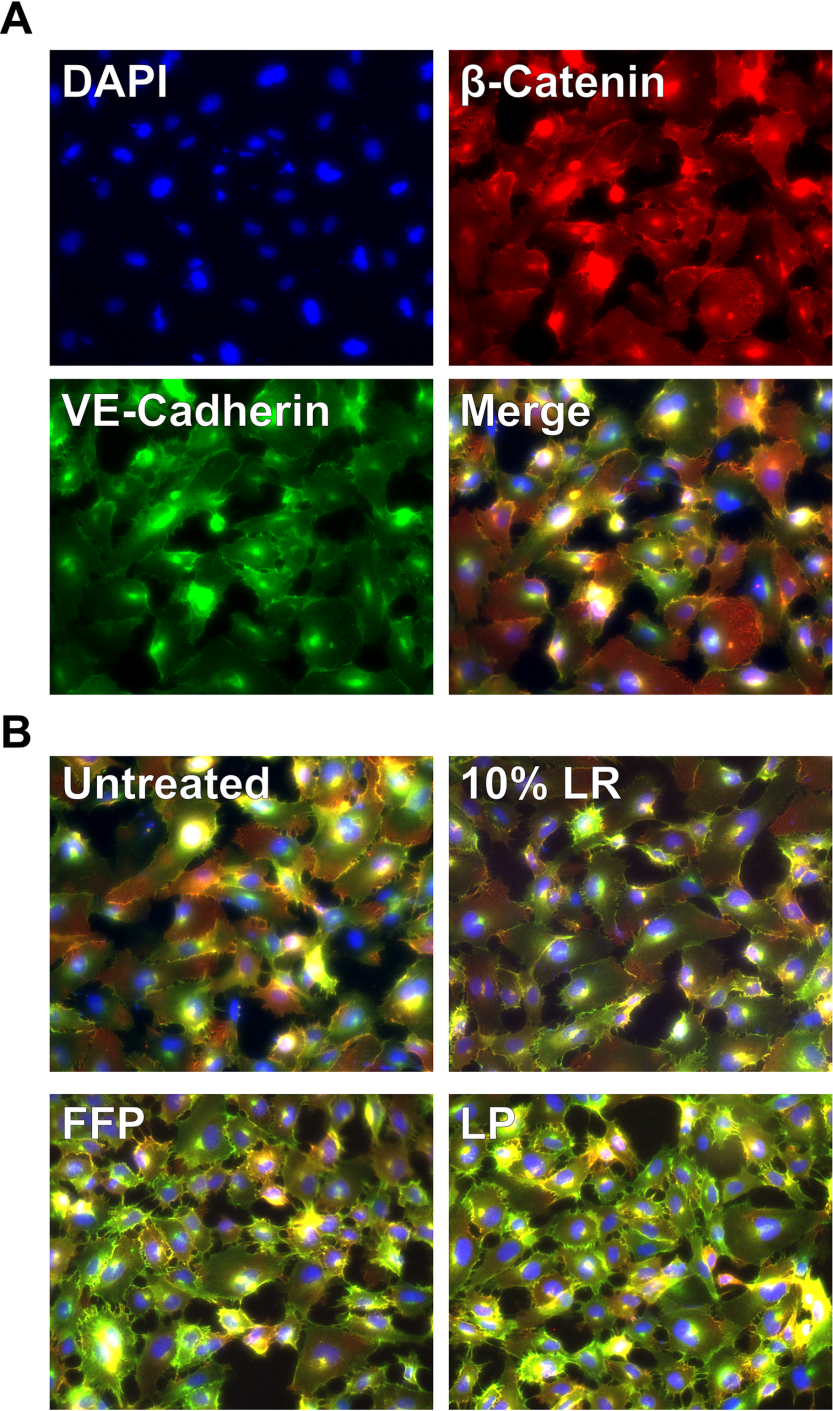
FFP and LP prevent VEGF disruption of adherens junctions. Cells were pretreated with LR, FFP, LP or not treated for 1 hour before VEGF was added. Cells were fixed and stain for (A) Dapi (blue), β-Catenin (red) and VE-Cadherin (green). In (B) it is qualitatively observed that FFP and LP preserve the overlap of VE-Cadherin and β-Catenin (yellow) compare to untreated and LR controls.

To further understand at the molecular level the effects of LP and FFP on endothelial junctional stability, we investigated the adherens junctions. Adherens junctions are critical regulators of vascular endothelial junctional stability and composed of beta-catenin and VE-cadherin. [20,33–36]. Monolayers stimulated with VEGF-A, an instigator of AJ breakdown and paracellular permeability, were stained for both beta-catenin and VE-Cadherin (Fig 3). FFP and LP both qualitatively restored adherens junction integrity as depicted by enhanced staining pattern of VE-Cadherin (green) and beta- catenin (red). Neither LR nor media alone had any notable effects on preventing adherens junction breakdown by VEGF-A.

### FFP and LP attenuate endothelial cell-leukocyte binding

**Fig 4:**
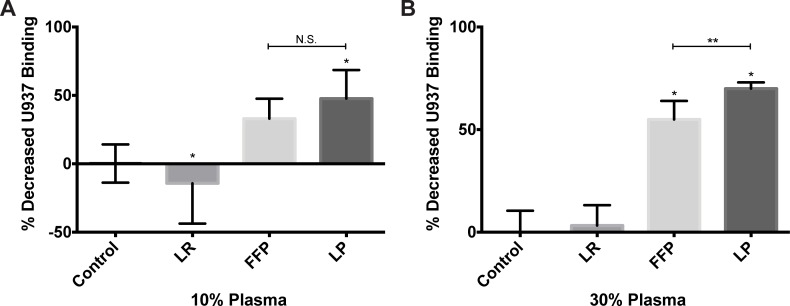
LP and FFP reduce that amount of leukocyte binding in vitro. Fluorescently labeled U397 cells were added to wells of HUVEC cells treated with 10(A) and 30(B) percent LR, FFP, LP or no treatment control. Unbound cells were removed after a period of one hour and the remaining cells quantified by fluorescence. All wells were normalized to control. * = (p<0.05) compared to control via post hoc turkey tests of an unpaired one-way ANOVA. N.S. clarifies that the indicated groups are Not Significantly different. Controls and FFP were featured in Wataha et al. (2013). [16].

We next sought to determine if plasma products modulated leukocyte binding to ECs. U937 cells, a monocyte cell line that we have used in the past on these studies of leukocyte-EC binding were utilized [16,33]. To stimulate U937 binding, ECs were treated with TNF-α then the relative binding of calcein-labeled U937 cells bound to ECs was quantified by fluorimetry. Binding studies revealed that treatment of the ECs with 30% and 10% LP significantly inhibited U937 binding to the ECs (Fig 4A and 4B) compared to media alone and LR as depicted by fold decrease in leukocyte binding. Only 30% FFP showed a significant inhibition of binding. Furthermore, there were no significant differences found between the effects of FFP and LP on leukocyte-EC adhesion at 10% but at 30% LP significantly decreased binding compared to FFP.

### LP reduces pulmonary vascular permeability, edema, and inflammation following HS

**Fig 5:**
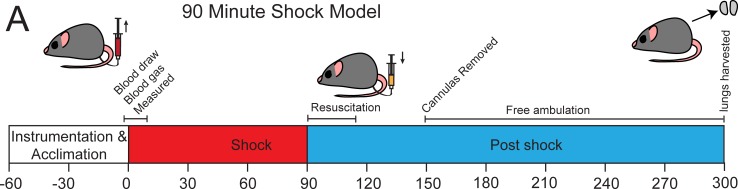
Schematic of hemorrhagic shock experiment. Animals, excepting shams, under went hemorrhagic shock for ninety minutes before being either being resuscitated with LR, FFP or LP, or receiving no intervention.

**Fig 6:**
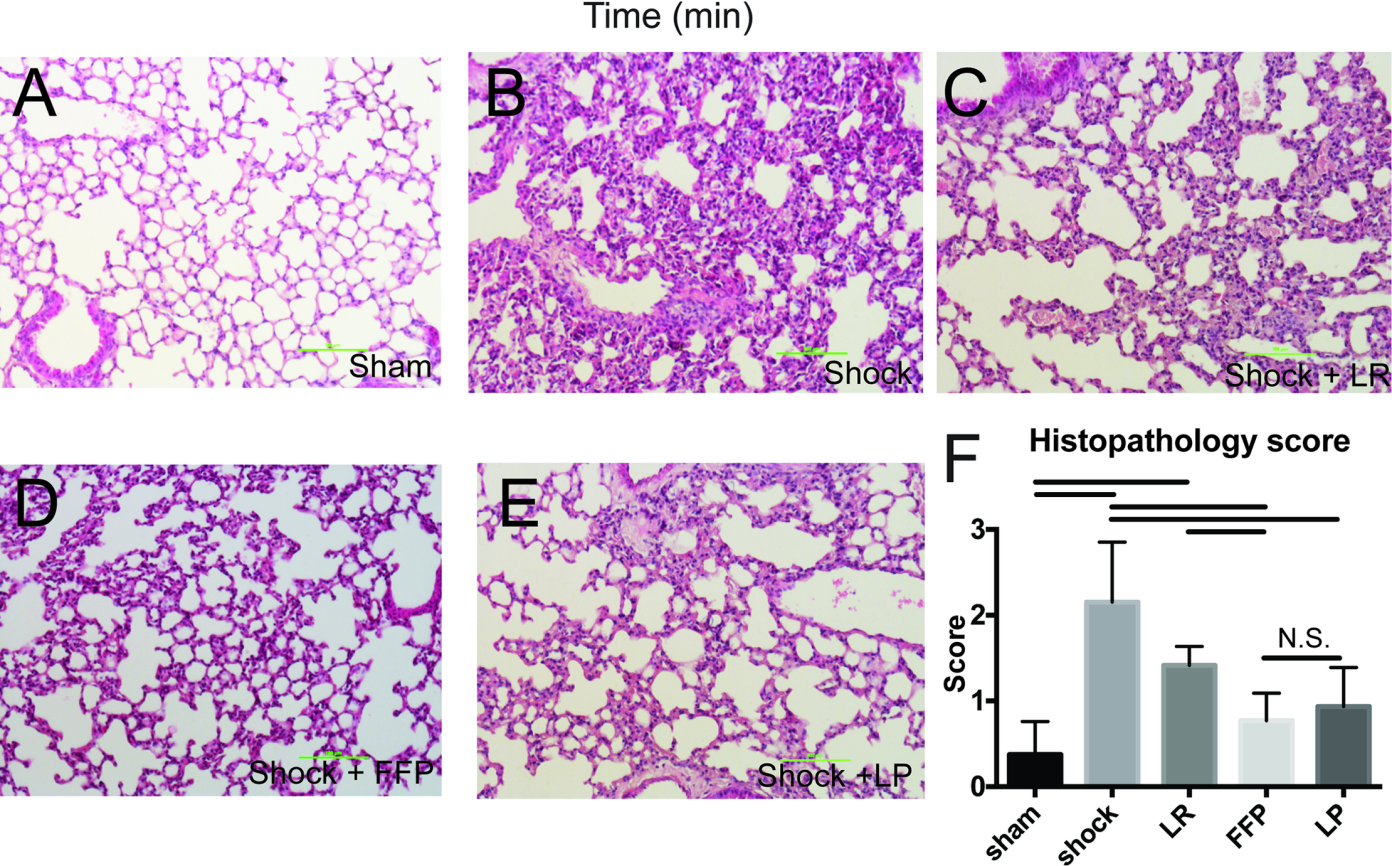
LP and FFP reduce the damage done to the lungs by a model hemorrhagic shock. Representational images of lungs stained with H&E from Sham (A), Shock (B), Shock+ LR (C), Shock + FFP (D) and Shock + LP (E). Histopathology scores average across all animals (F). Bars indicate significant differences (p < 0.05) via post hoc tukey tests based on a one-way ANOVA. N.S. clarifies that the indicated groups are Not Significantly different.

**Fig 7:**
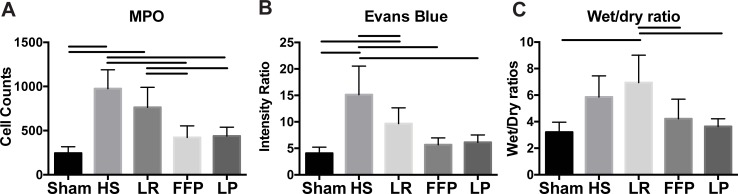
Measures of permeability and inflammation in the lungs after hemorrhagic shock. (A) Resuscitation with FFP and LP reduce the number of cells positive for MPO, a marker of inflammation compared to LR and shock alone. (B) Treatment with FFP or LP reduces the amount of Evans blue permeability to a point statistically indistinguishable from sham treatment after 3 days. (C) Both plasma groups also attenuate edema compared to animals treated with LR but not compared to HS alone. Bars indicate significant differences (p < 0.05) via post hoc tukey tests based on a one-way ANOVA.

To evaluate the ability of LP to reduce the HS-induced lung injury, we employed our established murine model of fixed pressure HS and laparotomy (Fig 5). [16,17,34] [12,13,25] We found that lung injury was significantly attenuated in LP and FFP treated mice as indicated by histopathological analysis (Fig 6A–6E) of H&E stained sections and calculated lung injury scores (Fig 6F). Myeloperoxidase (MPO) staining, an indication of neutrophil infiltration, revealed decreased MPO staining in the lungs from LP and FFP treated mice compared to LR and shock alone and was comparable to shams. (Fig 7A). In a separate set of experiments, HS mice treated with LP and FFP similarly decreased permeability and lung edema (Fig 7B and 7C). LP and FFP comparably decreased Evan’s Blue dye extravasation, indicative of permeability and decreased lung wet:dry ratios compared to LR and shock alone.

## Discussion

We have previously shown that fresh frozen plasma has potent protective effects on EC function and vascular barrier integrity *in vitro and in vivo*. [12,14,16,20] We now hypothesized that the stabilizing effects of LP on the endothelium *in vitro* and *in vivo* would be superior to Lactated Ringers solution and similar to our findings for FFP. We have indeed demonstrated that LP and FFP similarly inhibited EC permeability and trans-endothelial monolayer resistances, adherens junction reconstitution and leukocyte-EC binding *in vitro*. Minor differences between the two were found in that *in vitro*, LP is more effective at inhibiting leukocyte adhesion than FFP at the lower dose (10%). This is also the case for theability to inhibit permeability to dextran dye in endothelial monolayers. These difference may be manifesting in vitro since the donors of the LP are different than the FFP. However, these differences did not appear *in vivo* as FFP and LP equivalently mitigated pulmonary vascular permeability and edema, lung injury and inflammation induced by HS. Taken together, these studies support the central premise that LP can potentially be used as a substitute to FFP to correct endothelial dysfunction and mitigate the endotheliopathy of trauma (EOT). [9]

FFP resuscitation has also been shown to partially restore the damaged endothelial glycocalyx, and syndecan-1 expression. [13,15,20] Restoration of the glycocalyx physically reinforces the endothelial barrier and attenuates leukocyte-endothelial cell adhesion. [21] We have not examined the effect of LP on the glycocalyx but our current data suggests it would be equally restorative. Though we have focused our study on the lung, we have also shown that FFP mitigates gut injury and inflammation in our mouse hemorrhagic shock model.[29] Additionally, work by Alam and colleagues demonstrated that FFP decreased mortality, blood brain barrier compromise and cerebral edema in a swine model of traumatic brain injury. [35] Based on the findings of the current study, we anticipate that LP would also provide protection to HS and trauma induced gut and brain compromise. This is a future direction of ongoing investigations.

The current study focused on the effects of LP on the endothelium not coagulation. Others have demonstrated that LP has potent corrective effects on coagulopathy in pre-clinical trauma bleeding models, similar to FFP. Lyophilized or freeze dried plasma has also been shown to have similar beneficial effects in reducing inflammation and mortality after HS in large animal models. [35,37–40]

There are limitations of the current study. Both the FFP and LP were from single donors but as we have demonstrated, donor variability can be present within single donor units of plasma. [23] Another limitation of the study is the use of human plasma in mice. Using human plasma in mice affords us the opportunity to study the clinical product used in humans in pre-clinical models of hemorrhagic shock. We believe this to be of important translational benefit but xeno-incompatibility is a possible confounder. However, when we compared the use of mouse to human plasma in our murine model of HS we found a lack of specifies specific differences in pulmonary indices of injury, permeability or edema despite some differences in hemodynamic parameters. [41] While our model of trauma/hemorrhagic shock does result in coagulopathy as shown in our manuscript below by Peng et al. We did not access coagulation in the current study, we were focused on endothelial function and inflammation. However, in a study by Lee et al, full volume reconstituted LP retained on average 86% coagulation factor activity compared to fresh plasma and when used in 1:1 ratios with red blood cells demonstrated superior hemostatic efficacy compared to FFP.

In summary, our data support the concept that lyophilized plasma has potent systemic effects on the vascular endothelium and post-hemorrhagic shock lung and mitigates the endotheliopathy of trauma (EOT). Since there is a dire clinical need for dried plasma products in remote and austere settings, both in the military and civilian settings, these data support the premise that LP can be used in lieu of FFP in bleeding patients following severe traumatic injury.

## Supporting information

S1 File Data file for all figures. This file contains the raw data for all graphs contained in this manuscript.
(XLSX)
